# Therapeutic Outcome of MR-Guided High-Intensity Focused Ultrasound (MR-HIFU) in Solitary versus Multiple Uterine Fibroids

**DOI:** 10.3390/healthcare10081471

**Published:** 2022-08-04

**Authors:** Bernd Erber, Vincent Schwarze, Frederik Strobl, Alexander Burges, Sven Mahner, Sophia Samira Goller, Jan Rudolph, Jens Ricke, Bastian Oliver Sabel

**Affiliations:** 1 Department of Radiology, University Hospital, LMU Munich, Marchioninistr. 15, 81377 Munich, Germany; 2 Die Radiologie am Isarklinikum, Sonnenstr. 24-26, 80331 Munich, Germany; 3 Department of Obstetrics and Gynecology, University Hospital, LMU Munich, Marchioninistr. 15, 81377 Munich, Germany

**Keywords:** MR-guided high-intensity focused ultrasound, MR-HIFU, solitary uterine fibroids, multiple uterine fibroids, uterine fibroid symptom and quality of life

## Abstract

MR-guided high-intensity focused ultrasound (MR-HIFU) is an effective method for treating symptomatic uterine fibroids, especially solitary lesions. The aim of our study was to compare the clinical and morphological outcomes of patients who underwent MR-HIFU due to solitary fibroid (SF) or multiple fibroids (MFs) in a prospective clinical trial. We prospectively included 21 consecutive patients with SF (10) and MF (11) eligible for MR-guided HIFU. The morphological data were assessed using mint Lesion™ for MRI. The clinical data were determined using the Uterine Fibroid Symptom and Quality of Life (UFS-QOL) questionnaire before and 6 months after treatment. Unpaired and paired Wilcoxon-test and *t*-tests were applied, and Pearson’s coefficient was used for correlation analysis. A *p*-value of 0.05 was considered statistically significant. The volume of treated fibroids significantly decreased in both the SF (mean baseline: 118.6 cm^3^; mean 6-month follow-up: 64.6 cm^3^) and MF (107.2 cm^3^; 55.1 cm^3^) groups. The UFS-QOL showed clinical symptoms significantly improved for patients in both the SF and MF groups regarding concern, activities, energy/mood, and control. The short-term outcome for the treatment of symptomatic fibroids in myomatous uterus by MR-guided HIFU is clinically similar to that of solitary fibroids.

## 1. Introduction

Uterine fibroids are the most common benign neoplasms of the uterus in women of reproductive age, with a prevalence of 20–40% and the most common indication for a hysterectomy [1]. These estrogen-dependent tumors originate from the smooth muscle cells [2] and can occur in solitary or multiple forms in different locations in the uterus [3]. About 20–25% of uterine leiomyomata cause symptoms, and menstrual abnormalities are the most frequent [2]. The severity of menorrhagia seems to be increased for submucous lesions, although this symptom can also occur when fibroids are intramurally or subserously located [2]. Different reasons are found in the literature for menorrhagia. Some studies suggested that the decreased uterine contractility due to leiomyomata leads to menorrhagia [4], while others claimed that the dysregulation of local growth factors and aberrant angiogenesis might be relevant [5]. Furthermore, increased severity of urogenital symptoms was found for both anterior and intramural fibroids [6].

At present, myomectomy is the preferred treatment option. However, new interventional treatment options have emerged that offer benefits in terms of relieving symptoms while avoiding the risks of surgery: uterine artery embolization (UAE) and high-intensity focused ultrasound (HIFU) [7]. HIFU ablation can lead to comparable long-term outcomes with longer time intervals until re-intervention compared with myomectomy [8]. Furthermore, several studies compared the outcomes of HIFU and UAE. Reviews by Liu et al. [7] and Yan et al. [9] found the benefits of UAE regarding symptom relief and re-intervention rate. In their meta-analysis, Gao et al. found the lowest incidence of major complications with HIFU compared with UAE and myomectomy [10]. The technical effectiveness and safety of HIFU were reported in the literature for this relatively new treatment option [11]. Especially in solitary lesions, HIFU has been proven to be a valid method to improve clinical outcomes [12,13]. Therefore, solitary fibroids (SFs) are preferentially treated at many centers, although there is—to the best of our knowledge—no evidence in the literature on whether and to what extent patients with multiple fibroids (MFs) can benefit from MR-HIFU. Therefore, the aim of this study was to compare the clinical and morphological outcomes of patients with SF to those with MFs undergoing MR-HIFU treatment in a prospective clinical trial.

## 2. Materials and Methods

### 2.1. Patients

All participants underwent a contrast-enhanced MRI of the uterus in the prone position for treatment evaluation and were seen by a board-certified specialist in gynecology with more than 15 years’ experience and a board-certified specialist in radiology with more than 10 years’ experience in MR imaging as well as 5 years’ experience in MR-guided HIFU. The indication for HIFU ablation was interdisciplinarily made. The inclusion criteria were as follows: (1) the presence of solitary or multiple symptomatic uterine fibroids; (2) the patient’s written informed consent; (3) women aged over 18-years; (4) access to over 50% of the total fibroid volume for both solitary and multiple uterine fibroids. The exclusion criteria were as follows: (1) any contraindication of MRI contrast agent; (2) general contraindications regarding MRI safety; (3) any clinical or MR-morphological signs of malignancy such as leiomyosarcoma; (4) a positive pregnancy test; (5) bowel in the direct path of the HIFU beam in the pre-interventional MRI. Patients received contrast-enhanced imaging prior to treatment, immediately after treatment and at the 6-month follow-up.

### 2.2. MR-Guided HIFU Ablation

All patients were treated with the same commercially available MR-HIFU system (Sonalleve™, Profound Medical Corp., Mississauga, ON, Canada) integrated into a 3.0T MR scanner (Ingenia 3.0T, Philips, Amsterdam, The Netherlands). Details of the MR-HIFU system and respective treatment procedures have been described in the literature before [14,15]. Apart from those showing contraindications such as allergies, all patients intravenously received 1000 mg metamizole and 500 mg paracetamol as an analgesic and 1 mg lorazepam. Patient immobilization was limited to 3 h. In most cases of MFs, not all the fibroids were accessible for HIFU treatment, e.g., when the distance between the skin and lesion was too large. In these patients, the aim was to treat fibroids that were considered to be symptomatic, e.g., larger and anteriorly or intramurally located lesions that might cause urogenital symptoms such as frequent urination [6]; or lesions that were submucosally located, thus posing a higher risk of menorrhagia [2].

### 2.3. MRI Data Acquisition and Postprocessing

#### 2.3.1. Data Acquisition

The MRI studies were performed with a 1.5 T magnet (Avanto; Siemens Healthineers; Erlangen; Germany or Ingenia 3.0T, Philips, Amsterdam, The Netherlands). Immediate pre-interventional MRI was performed on the day of intervention for planning HIFU ablation, and consisted of T2-weighted (T2w) and T1-weighted sequences without contrast. The immediate post-intervention MRI consisted of contrast-enhanced T1-weighted fat-saturated (T1w fs CE) sequences. The pre-intervention baseline imaging for the evaluation of HIFU indication and the imaging for the 3-month follow-up consisted of T1- and T2-weighted multiplanar sequences as well as contrast-enhanced T1-weighted fat-saturated sequences.

#### 2.3.2. MRI Postprocessing

All the MRI data were transferred and post-processed using mint Lesion™ (Mint Medical GmbH, Heidelberg, Germany). A resident with 3 years’ experience in MR imaging manually segmented the fibroid margins in T2w and T1w fs CE sequences and a nonperfused volume (NPV) using T1w fs CE sequences with volumetric measures using mint Lesion™. The signal intensity (SI) of the fibroids was measured in both T2w and T1w fs CE sequences and was set in relation to the SI of the healthy areas of the uterus and skeletal muscles. An assessment of the fibroid localization (intramural, submucous, subserous) was made by the consensus of a board-certified specialist with more than 10 years’ experience and a resident with more than 3 years’ experience in MR imaging.

### 2.4. Disease-Specific Symptom and Health-Related Quality of Life Questionnaire for Leiomyomata

The Disease-Specific Symptom and Health-Related Quality of Life Questionnaire for Leiomyomata (UFS-QOL) was given to patients before and 6 months after the HIFU ablation; this is a validated instrument consisting of 8 symptom- and 29 health-related quality of life items [16]. The questionnaire was designed to assess symptom severity and symptom impact on the quality of life for women with leiomyomata. It was shown to differentiate leiomyomata patients with varying degrees of symptom severity [16]. The UFS-QOL has been applied several times in various studies [17,18,19]. Items are labeled as concern (5 items; minimal score: 5; maximal score: 25), activities (7; 7; 35), energy/mood (7; 7; 35), control (5; 5; 25), self-consciousness (3; 3; 15), and sexual function (2; 2; 10). We combined self-consciousness and sexual function because these two groups consisted of a smaller number of items in comparison with the other groups.

### 2.5. Statistical Analysis

A statistical analysis was performed using the open-source programming language R (Version 4.0.4, RStudio Inc., Boston, MA, USA) [20]. The differences in continuous morphologic variables were compared using paired and unpaired *t*-tests. Categorial variables regarding UFS-QOL were compared using paired and unpaired Wilcoxon tests for nonparametric data. For the correlation analysis of numeric data, the Pearson correlation coefficient was used. A *p*-value of 0.05 was set as the limit of statistical significance.

## 3. Results

### 3.1. Patients

The mean age for patients with SF was 43.9 years (min 35.0; max 54.0) and 44.8 years (33.0; 52.0) for patients with MFs.

The SFs were submucous in four cases and intramural in six cases, while in the eleven patients with MF, treated lesions were submucosally located in seven cases, subserosally in one case and intramurally in nine cases.

Regarding Funaki’s classification (21), three fibroids were assessed to be grade 1, five were grade 2 and two were grade 3 in the SF group. Among the patients with a myomatous uterus, seven fibroids were assessed with Funaki grade 1, eight fibroids as grade 2, and one fibroid as grade 3.

The relative SI of the SF and MF groups showed the same mean value of 2.0.

**Figure 1:**
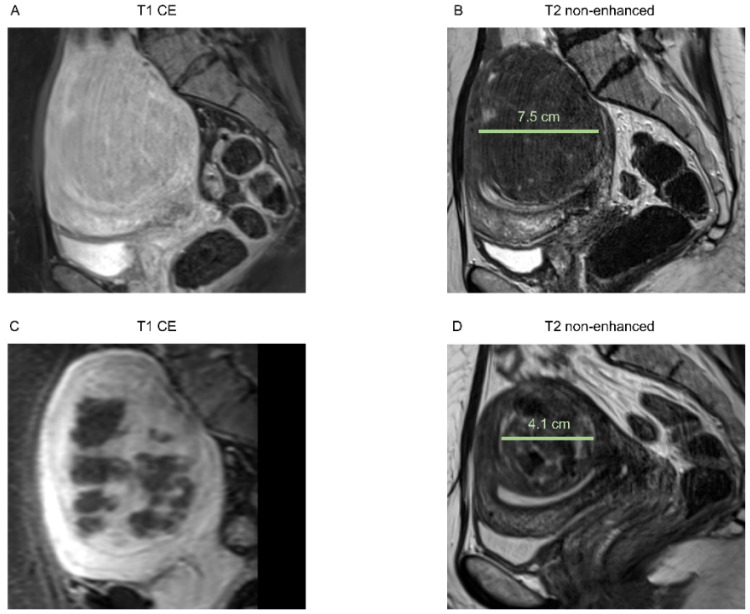
Images of a 51-year-old patient with solitary intramural uterine fibroid in the posterior wall, Funaki grade 2. (**A**) Contrast-enhanced T1-weighted and (**B**) T2-weighted images 2 weeks before HIFU treatment, sagittal reformation. Pre-interventional maximum diameter was 7.5 cm. (**C**) Immediate post-interventional T1 contrast-enhanced image with a volumetric assessed NPV of 61%, sagittal reformation. (**D**) T2-weighted image 6 months after intervention with a maximum diameter of 4.1 cm, sagittal reformation.

**Figure 2:**
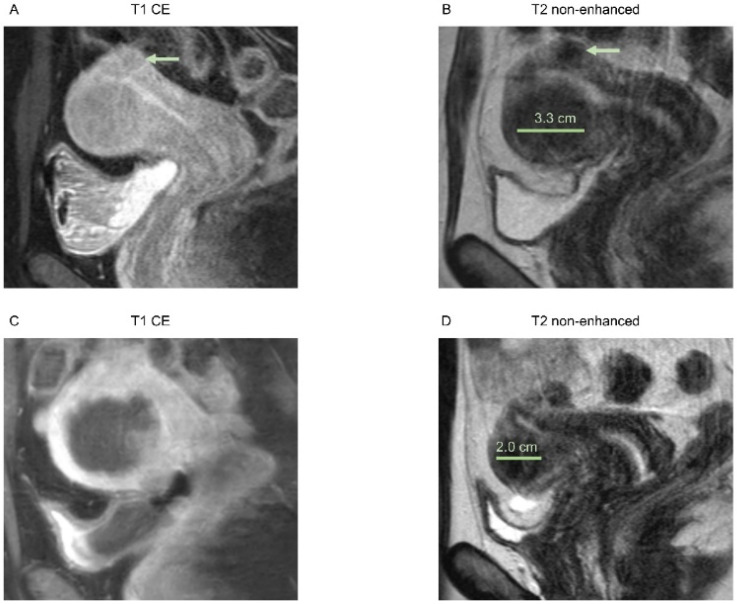
Images of a 39-year-old patient with multiple uterine fibroids in the anterior wall and posterior wall, Funaki grade 2. (**A**) Contrast-enhanced T1-weighted and (**B**) T2-weighted images 3 weeks before HIFU treatment of the fibroid in the anterior wall, sagittal reformation. Pre-interventional maximum diameter was 3.3 cm. A smaller subserous fibroid is depicted in the posterior wall (arrow), which could not be reached by HIFU due to bowel loops in the beam path. (**C**) Immediate post-interventional T1 contrast-enhanced image with a volumetric assessed NPV of 64%, sagittal reformation. (**D**) T2-weighted image 6 months after intervention with a maximum diameter of 2.0 cm, sagittal reformation.

In Figure 1 and Figure 2, examples of an MRI of both groups are shown.

### 3.2. Morphological Assessment

**Figure 3:**
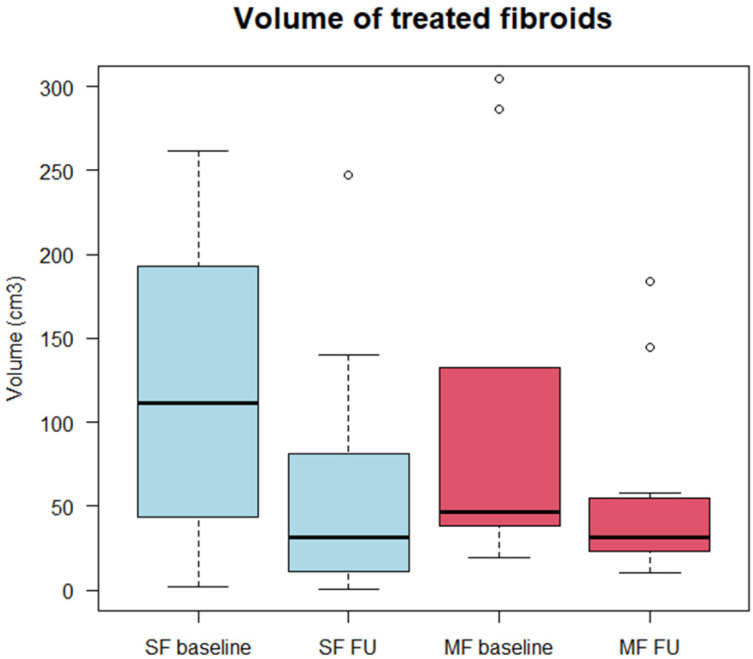
Boxplots showing minimum, first quartile, median, third quartile, and maximum for baseline and 6-month follow-up (FU) volume in centimeters cubed for treated solitary (SF) and multiple (MFs) fibroids. Differences between baseline and 6-month FU were significant for both SF and MF groups.

The mean volume of treated SFs was 118.6 cm^3^ (min 2.3; max 261.8; SD 98.4) and 107.2 cm^3^ (19.3; 304.5; 101.3) for treated lesions in patients with MFs. For patients with MFs, the whole fibroid mass (volume of treated and untreated fibroids) was 152.7 cm^3^ (22.4; 460; 153.5). In the 6-month follow-up, the volume of treated fibroids significantly decreased in both groups: for SFs, the mean volume in the 6-month follow-up was 64.6 cm^3^ (0.5; 247.2; 77.4), while for treated MFs, it was 55.1 cm^3^ (10.0; 183.7; 56.3). The data are visualized in Figure 3.

This resulted in a relative fibroid volume reduction of 52.2% for SF (min −0.1%; max 83.9%; SD 0.31) and 45.5% for MFs (14.6%; 76.9%; 15.8%) compared with the baseline. No statistically relevant difference was found in the relative volume reduction between both groups (*p* = 1).

The mean NPV ratio for SF was 0.56 (0.41; 0.79; 0.10) and 0.64 (0.39; 0.73; 0.10) for MFs. No statistically relevant difference was found in the NPV between the groups (*p* = 0.12).

The correlation between the baseline volume of treated fibroids and relative volume reduction was very low (r = 0.05). No relevant correlation was found between the relative baseline SI of fibroids and relative volume reduction (r = 0.07).

### 3.3. Symptom Severity (UFS-QOL)

For patients with SF, the mean score for concern (five items; minimal score: 5; maximal score: 25) was 17.0 (min. 11.0; max. 25.0; SD 5.2) and 12.8 (min. 6.0; max. 23.0; SD 7.0) for patients with MFs. Regarding activities (7; 7; 35), the mean score for patients with SF was 21.9 (14.0; 33.0; 6.4) and 18.1 (9.0; 30.0; 7.4) for patients with MFs. The mean score for energy/mood (7; 7; 35) was 21.8 (10.0; 35.0; 9.0) for SF and 18.9 (10.0; 31.0; 6.3) for MFs. The baseline score for the control (5; 5; 25) was 16.5 (11.0; 23.0; 4.3) for the SF patients and 13.5 (7.0; 22.0; 5.2) for the MF patients. On self-consciousness and sexual function (5; 5; 25), the mean for the SF patients was 12.5 (7.0; 21.0; 4.4) and 12.1 (5.0; 21:0; 4.5) for the MF patients. The baseline scores for concern and activities significantly differed between the SF and MF patients, while differences in the other three groups were not statistically significant.

In the 6-month follow-up, the mean score for concern significantly decreased for the SF patients from 17.0 to 11.8 (6.0; 22.0; 4.8) and from 12.8 to 7.5 (5.0; 16.0; 3.6) for the MF patients. The mean score for activities significantly decreased from 21.9 to 14.7 (7.0; 22.0; 5.0) for the SF patients and from 18.1 to 11.7 (7.0; 20.0; 4.8) for the MF patients. For patients with SF, the mean score for energy/mood significantly decreased from 21.8 to 14.4 (7.0; 24.0; 5.5) and, for patients with MFs, from 18.9 to 12.0 (7.0; 20.0; 3.9). Additionally, mean scores for control significantly decreased from 16.5 to 10.8 (6.0; 19.0; 3.9) for SF and from 13.5 to 8.6 (5.0; 15.0; 3.3) for MFs. Finally, for self-consciousness and sexual function, the mean score for the SF patients significantly decreased from 12.5 to 9.2 (6.0; 14.0; 2.5) and from 12.1 to 8.3 (5.0; 19.0; 4.7) for the MF patients. The decrease for the MF patients in self-consciousness and sexual function was not significant (*p* = 0.10).

Compared with the baseline scores, we found a relative decrease in symptom severity in the 6-month FU regarding concern to 70.8% for patients with SF and 68.0% for patients with MFs; regarding activities, to 69.1% for SF patients and 69.4% for MF patients; regarding energy, to 69.5% for SF patients and 68.2% for MF patients; regarding control, to 66.6% for SF patients and 69.3% for MF patients; regarding self-consciousness and sexual function, to 78.3% for SF patients and 75.8% for MF patients.

**Figure 4:**
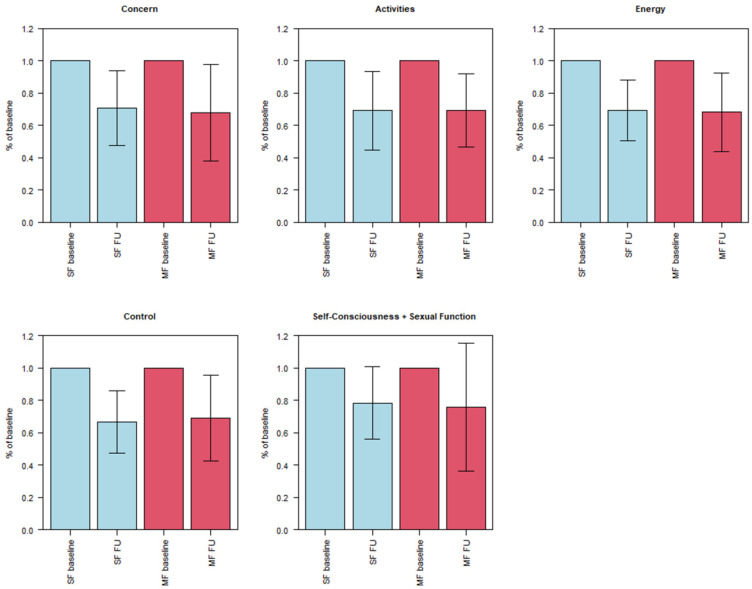
Boxplots showing minimum, first quartile, median, third quartile and maximum for scores of UFS-QOL items: concern, activities, energy/mood, control and combined self-consciousness and sexual function. Both scores of 6-month FU were normalized to baseline scores. Differences between baseline and 6-month FU were significant for both SF and MF groups regarding concern, activities, energy/mood, and control. No significant difference was found for the MF group in self-consciousness and sexual function.

The data are visualized in Figure 4.

## 4. Discussion

The aim of our study was to compare the clinical and morphological outcomes of patients with solitary fibroids with those of patients with multiple fibroids who underwent MR-HIFU treatment in a prospective clinical trial. The volume of treated fibroids significantly decreased in both the SF and MF patients after treatment. Clinical symptoms significantly improved for most UFS-QOL items. No significant difference was found between groups regarding the improvement in symptoms.

Our findings agree with those in the current literature showing that HIFU is an effective method to treat symptomatic uterine fibroids. The relative volume reduction of treated fibroids for all 21 patients was between 46% and 52% after 6 months. This corresponds to the findings of Chang et al. [12], who found a relative volume reduction of 32.8–44.1% for solitary fibroids after 6 months. In our study, the relative volume reduction was slightly higher for SF (52.2%) than for treated MFs (45.5%); however, the differences were not statistically significant. Nevertheless, this is an interesting result, as the relative SI of treated fibroids nearly was the same in both groups. According to Funaki’s classification [21], fibroids with a lower SI are more suitable for HIFU than fibroids with a higher SI, as a higher SI is associated with hypervascularity and, therefore, a lower response to HIFU. Additionally, we did not find a relevant correlation between the relative SI of the fibroids and relative volume reduction. However, the discrepancy in the relative volume reduction could not be explained by different treatment characteristics, as the NPV—which was shown to be a relevant factor for the outcome of HIFU therapy [22]—was similar in both groups. In our study, the mean baseline volume of SF was slightly higher than that of the treated fibroids for patients with MF. Chang et al. [12] found a higher impact of HIFU therapy on patients’ symptoms as well as a reduction in fibroid volume for lesions that were smaller than 10 cm in diameter. Cheng et al. [23] found significantly higher ablation rates for fibroids with diameters greater than 7 cm compared to smaller lesions (<3 cm or 5–7 cm). However, these results may not translate to a comparison between solitary and multiple fibroids. As described in the Results section, the correlation between the baseline fibroid volume and relative volume reduction was very low. In summary, SF seem to respond slightly better to HIFU therapy than MFs regarding relative volume reduction. However, the differences between the groups were not statistically significant.

More relevant for clinical efficiency and patient sufficiency is the post-interventional symptom severity. Interestingly, the baseline scores for all items were slightly higher for SF than for MFs, although differences were not significant. This might be explained by the difference in baseline fibroid volume, which was higher for SF than for MFs. The improvement in symptom severity was, except for self-consciousness and sexual function, significant for all items for patients with either solitary or multiple fibroids. This is remarkable as improvement in symptom severity is the most relevant outcome parameter for patients suffering from this benign uterine neoplasm.

As described earlier, for patients with MFs, not all lesions were feasible for HIFU therapy, e.g., because they were not accessible for the HIFU beam due to a large distance to the skin, as described in the Methods section. However, the finding that patients with MF had a similar symptomatic benefit to those with SF supports the hypothesis that treatment of symptomatic fibroids in patients with MF is essential for clinical outcomes. It is expected that submucous fibroids frequently cause menorrhagia, although this symptom is also seen with fibroids in other locations. However, the severity of bleeding seems to be increased by the presence of submucous fibroids [2]. For the severity of dysuria, an increase was found for anterior myomas compared with other locations [6,24]; additionally, an intramural location seemed to be associated with increased urinary symptoms [2,25].

Symptom severity (UFS-QOL) was assessed in a short-term follow-up after 6 months. In both the SF and MF groups, a distinct and statistically significant improvement in symptom severity was found, which was accompanied by significant fibroid volume reduction. This is in line with the findings in the literature, for example, Kim et al., who found significant improvement in UFS-QOL scores after 3 months, which was sustained sustainability for a period of 3 years [26], or Chang et al. [12], who described a significant benefit after 6 months.

An interesting question for further study is a comparison between HIFU and UAE for the outcomes of patients with MFs. UAE may have promising results for lesions in patients with MFs that are not accessible for HIFU and may be addressed by this method [27].

### Limitations

The inclusion of follow-up examinations only after 6 months is a limitation of the study. For this reason, further studies are needed to compare HIFU treatment for SF and MFs at later follow-up, especially because, in most cases of MFs, not all lesions are accessible or feasible for HIFU treatment and, therefore, might be prone to further growth. Therefore, a longer follow-up is needed to analyze the possible recurrence of symptoms.

Another limitation is the limited number of patients in both groups, as MR-guided HIFU is a novel treatment option and the study was designed as a single-center trial. Hence, we were unable to obtain more patient data. However, the results show a clear and promising benefit of MR-HIFU therapy for patients with multiple fibroids.

## 5. Conclusions

The short-term outcome for the treatment of symptomatic fibroids in myomatous uterus with MR-guided HIFU is clinically and morphologically similar to that for solitary fibroids. Therefore, MR-guided HIFU therapy should be considered for patients with multiple lesions. Further long-term follow-up is needed to analyze the sustainability of symptom relief.

## Data Availability

The data presented in this study are available on request from the corresponding author.
